# Turnover intention and its predictors among Emergency Medical Services (EMS) professionals: a systematic review and meta-analysis

**DOI:** 10.1186/s13049-026-01567-8

**Published:** 2026-01-26

**Authors:** Ehsan Zarei, Mehdi Safari, Zahra Zamani, Edris Kakemam

**Affiliations:** 1 Research Institute for Health Sciences and Environment, Shahid Beheshti University of Medical Sciences, Tehran, Iran; 2 Department of Disasters and Emergencies, School of Public Health and Safety Shahid, Beheshti University of Medical Sciences, Tehran, Iran; 3 Department of Health Policy and Management, School of Public Health and Safety, Shahid Beheshti University of Medical Sciences, Tehran, Iran; 4 Non-Communicable Diseases Research Center, Research Institute for Prevention Non-Communicable Diseases, Qazvin University of Medical Sciences, Qazvin, Iran

**Keywords:** Turnover intention, Intention to leave, Pre-hospital emergency services, Paramedic, Emergency Medical Services (EMS)

## Abstract

**Background:**

High turnover intention among pre-hospital Emergency Medical Services (EMS) professionals threatens the sustainability of these vital health services. This study aimed to determine the global prevalence of turnover intention and identify its predictive factors to inform effective retention strategies.

**Methods:**

This systematic review and meta-analysis followed the PRISMA 2020 guidelines. The PubMed, Web of Science, and Scopus databases were comprehensively searched up to August 31, 2025. Observational studies reporting on turnover intention and its associated factors were included. The pooled prevalence was calculated using a random-effects model, and influencing factors were analyzed within the expanded Job Demands-Resources (JD-R) framework.

**Results:**

From 2,077 identified records, 27 studies with an overall sample size of 129,580 participants were eligible for the systematic review. A meta-analysis of 19 studies revealed a global pooled prevalence of turnover intention of 23.5% (95% CI: 16.6%-32.1%). The prevalence was significantly higher in studies with small sample sizes (36.4%) compared to those with large sample sizes (11.7%) (*p* < 0.001). The pooled prevalence also differed significantly across North America (13.4%), Europe (31.6%), and other regions (45.7%) (*p* < 0.001). Job dissatisfaction and job stress were the most frequently reported predictors, followed by burnout, high workload, inadequate compensation, and poor physical and mental health.

**Conclusion:**

Approximately one in four EMS professionals worldwide intend to leave their job. This phenomenon is a response to the imbalance between high job demands and inadequate resources. Retaining this critical workforce requires a dual approach: strengthening resources at the organizational level and implementing structural reforms at the macro-policy level.

**Supplementary Information:**

The online version contains supplementary material available at 10.1186/s13049-026-01567-8.

## Introduction

Emergency Medical Services (EMS) represent a critical pillar of pre-hospital care, annually serving millions of patients, from trauma victims to individuals with chronic illnesses [1]. The efficiency and sustainability of this system, however, are threatened by a growing shortage of specialized professionals, a challenge largely rooted in high rates of job turnover [2, 3]. The nature of the EMS profession is inherently stressful, unpredictable, and demanding. Long shifts, repeated exposure to traumatic scenes, intense emotional pressures, and physical hazards make it one of the most challenging occupations in the healthcare sector [4]. Consequently, EMS professionals are significantly more susceptible to stress, burnout, post-traumatic stress disorder (PTSD), and other mental health issues compared to other healthcare workers [5].

Each year, despite substantial investments in training, a significant percentage of EMS professionals permanently leave the profession. This phenomenon has escalated into a global crisis, seriously threatening the stability and quality of pre-hospital services. The recruitment and retention of skilled professionals in EMS are challenging not only due to high replacement costs but also because they lead to a temporary reduction in the overall system's productivity [5, 6]. The turnover rate among paramedics has been increasing globally, with this trend intensifying over the last decade. This crisis is particularly acute in the United States, where the turnover rate reached 25% to 30% in 2022. Although lower rates are reported in countries such as the United Kingdom (approximately 10%) and Australia (4.1%), the intention to leave the profession remains high among personnel in these regions [7].

The departure of experienced staff from the EMS system results in multifaceted and detrimental consequences, including reduced quality of care, an increase in medical errors, substantial costs for recruiting and training new staff, and the loss of professional knowledge critical for mentoring new professionals. This pressure intensifies the workload for remaining paramedics and, by increasing ambulance response times, directly and adversely affects patient outcomes. The crisis is felt more severely in underserved areas where access to healthcare services is already limited. As the demand for emergency services grows due to factors like an aging population and a rise in chronic diseases, the shortage of specialized professionals jeopardizes the system's sustainability [5, 7, 8].

One of the most significant predictors of job turnover is Turnover Intention (TI), an individual's conscious willingness to leave their current organization or profession due to factors such as dissatisfaction [9–11]. Studying this "intention" is particularly important because, at this stage, no practical action to depart has yet been taken, providing an opportunity for effective interventions to retain the workforce [12]. High employee turnover can ultimately lead to attrition, a state where an organization is unable to fill vacant positions, resulting in a decrease in the overall size of the workforce [13].

The factors influencing job turnover in this group are multidimensional and complex, encompassing a range of individual and organizational issues. These include inadequate salary and benefits, unfavorable retirement conditions, a lack of career advancement opportunities, weak organizational support, ineffective leadership, and inflexible working conditions. Combined with a diminished sense of meaning and a lack of control over the work environment, these factors gradually lead to disillusionment and the decision to leave the profession, highlighting the necessity of developing targeted retention strategies [2, 5, 7, 14].

Identifying the factors that drive EMS professionals to leave their profession is of critical importance for healthcare organizations and policymakers. Despite numerous studies on job turnover in other health sectors, comprehensive research that investigates the reasons for turnover among paramedics using effective policymaking tools like systematic reviews and meta-analyses is limited. This research gap underscores the need for an integrated, in-depth analysis. Accordingly, this study was designed to conduct a systematic review and meta-analysis to determine the prevalence of turnover intention and identify its key associated factors among pre-hospital emergency professionals.

## Methods

### Study design

This study is a systematic review and meta-analysis, designed and reported following the "Preferred Reporting Items for Systematic Reviews and Meta-Analyses" (PRISMA 2020) statement [15]. The primary research question was: "What is the global prevalence of turnover intention among pre-hospital emergency professionals, and what are its predictive factors?".

### Information sources and search strategy

A comprehensive and systematic search was conducted in the PubMed, Web of Science (WoS), and Scopus databases for literature published up to August 31, 2025. To maximize the search's comprehensiveness and retrieve all potentially relevant studies, Google Scholar and the reference lists of selected articles were also manually screened (hand-searching). The search strategy was developed in consultation with an expert librarian, using a combination of the following keywords with Boolean operators (see Appendix S1):

("personnel turnover" OR "intention to quit" OR "intention to leave" OR "turnover intention") AND ("emergency responders" OR "paramedic" OR "EMT paramedics" OR "emergency medical technicians" OR "first responders" OR "ambulance crew" OR "Emergency Medical personnel" OR "Emergency Medical professional" OR "Ambulance Personnel" OR "EMS Providers" OR "Prehospital Emergency Technicians").

In cases where the full text of articles was unavailable, the corresponding author was contacted.

### Study selection

All retrieved articles were imported into EndNote reference management software (Version 20), where duplicates were identified and removed. In the next stage, two researchers independently screened the titles and abstracts of the remaining articles based on the eligibility criteria. Subsequently, the full texts of potentially relevant articles were retrieved and reviewed for final assessment. Any disagreements between the two researchers during the selection process were resolved through discussion and consensus, or if necessary, by consulting a third researcher.

### Eligibility criteria

To be included in this systematic review, studies had to meet the following criteria:

- Study Type: Original observational studies, including cross-sectional, longitudinal, and correlational designs. Review articles, letters to the editor, case reports, and other non-research studies were excluded.
- Population: Active personnel in pre-hospital emergency services, such as EMTs, Paramedics, EMS Professionals, Ambulance Personnel, or EMS Clinicians. To ensure consistency, this manuscript will collectively refer to this population as "EMS professionals".
- Outcome: Reporting "Turnover Intention" as a prevalence (percentage) or as quantitative data (mean and standard deviation). Studies solely reporting "Actual Turnover" were excluded.
- Publication Period: Studies published between 2000 and 2025.

### Data extraction

A standardized form was designed for systematic information extraction. Two researchers independently extracted key data from each eligible article, including: a) general study characteristics (first author, publication year, country); b) methodological features (study design, outcome measurement tool, sample size); and c) primary results (prevalence of turnover intention and its predictors).

### Risk of bias assessment

The methodological quality and risk of bias of selected studies were assessed using the Joanna Briggs Institute (JBI) Critical Appraisal Checklist for Analytical Cross-Sectional Studies [16]. This tool consists of eight items, each answered with "Yes," "No," "Unclear," or "Not Applicable." The assessment was performed independently by two reviewers, and disagreements were resolved through discussion or by involving a third reviewer. As no established cut-off point exists for this tool, a quality threshold of at least six "Yes" responses out of the eight possible questions was considered for inclusion in the meta-analysis.

### Data synthesis and analysis

Data synthesis was performed in two parts: quantitative and qualitative:

- Quantitative Analysis (Meta-analysis): All statistical analyses were conducted using Comprehensive Meta-Analysis (CMA) software (Version 3) based on a random-effects model. Statistical heterogeneity among studies was evaluated using the *I*^*2*^ statistic. A one-study-removed sensitivity analysis was performed to examine the robustness of the results. The likelihood of publication bias was assessed visually with a funnel plot and statistically with Egger's regression test (*p* < 0.10). Subgroup analyses were conducted based on factors such as the COVID-19 pandemic period, geographical region, country income level, and primary study sample size.
- Qualitative Analysis (Narrative Synthesis): A narrative synthesis approach was employed to integrate findings related to the predictive factors of turnover intention. The analytical framework for this section was based on the expanded Job Demands-Resources (JD-R) model [17], which categorizes influencing factors into three main domains: "Job Demands," "Job Resources," and "Personal Resources and Demands." The analysis used directed content analysis [18], where each factor extracted from the studies was coded and systematically assigned to one of the three predefined model categories. The findings were then narratively synthesized and presented, focusing on the reporting frequency and significance of each factor across studies.

## Results

### Study selection and characteristics

**Fig. 1:**
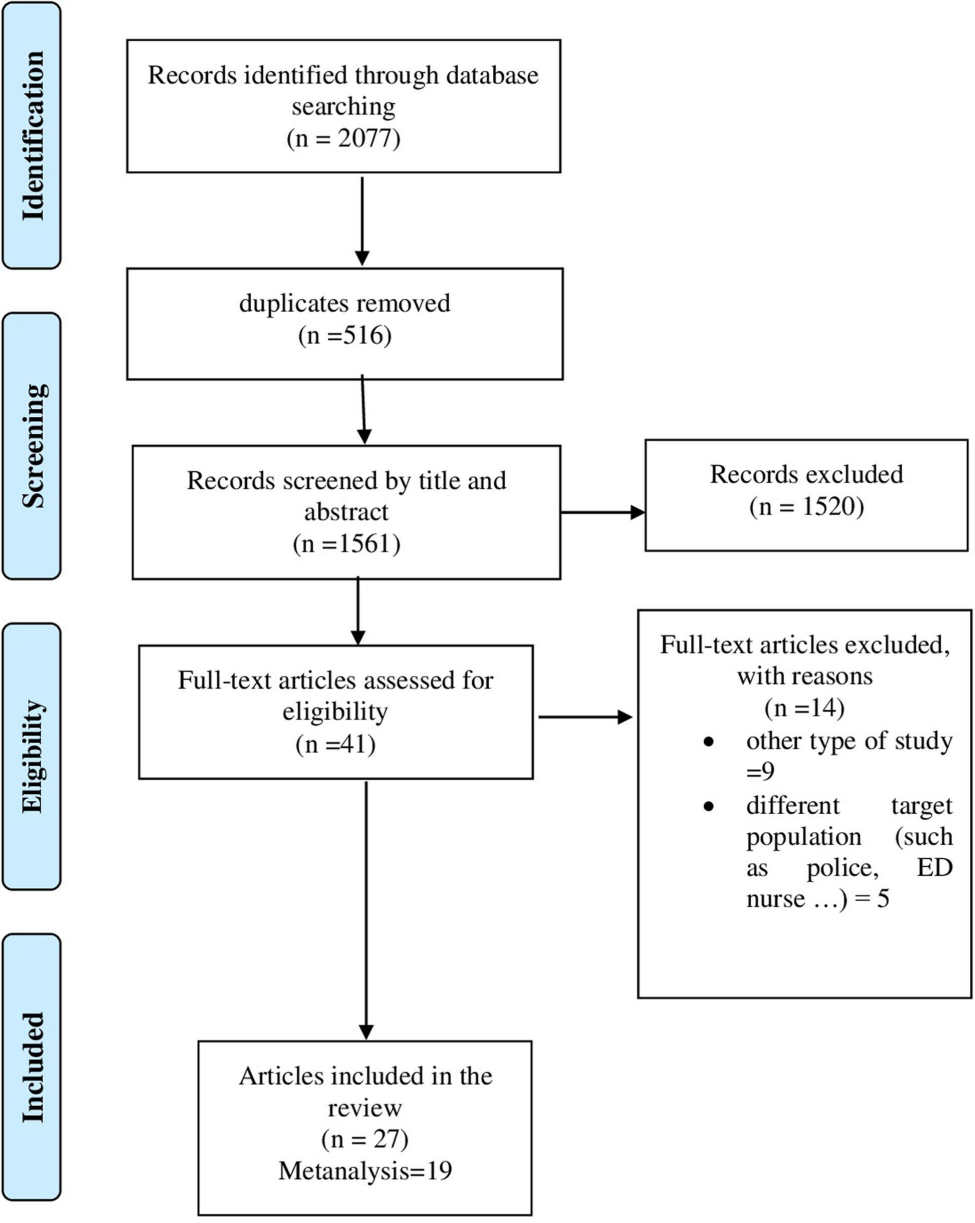
PRISMA flow diagram

The study selection process is detailed in the PRISMA flow diagram (Fig. 1). The initial search identified 2,077 articles. After removing duplicates and screening titles and abstracts, the full texts of 41 articles were assessed for eligibility. Ultimately, 27 studies meeting all pre-determined criteria were selected for the systematic review, of which 19 were eligible for the meta-analysis.

The 27 included studies comprised 129,580 pre-hospital emergency professionals. Sample sizes of individual studies varied considerably, ranging from 105 to 33,414 participants. The studies were conducted between 2009 and 2025 across eight different countries. The United States contributed the largest number of studies (*n* = 14), followed by Finland (*n* = 4), and Australia, Iran, and Germany (*n* = 2 each). One study each from South Africa, Romania, and Turkey was also included (Table 1).

**Table 1:** Sum﻿mary of included studies

	Author (year)	Country	Sample	TI (%)	TI Mean (SD)	Factors affecting TI
1	Blau (2009) [19]	USA	854 EMTs & paramedics	NR	1.92 (1.15)	• Low job satisfaction • Low organizational commitment • Low self-report health • Proportion of emergency to scheduled transports
2	Chapman (2009) [20]	USA	933 EMTs & paramedics	NR	1.65 (0.86)	• Poorer self-reported health • Lower Job satisfaction • Type of Transports (lower proportion of emergency calls) • Firefighter Training (Not being trained as a firefighter) • Lower earnings • higher number of work-related absences
3	Perkins (2009) [21]	USA	1008 EMTs	9%	NR	• Age (50 years of age or older) • Years of Service (10 or more years) • Low job satisfaction • Career change • Personal or family issues • Organizational issues • Work hours • Job stress • Pay/benefits
4	Bria (2013) [22]	Romania	105 ambulance personnel	30.5%	NR	• Burnout • Workload
5	Iwu (2013) [23]	South Africa	122 EMS personnel	21.1%	NR	• lack of promotional opportunities • Work Enjoyment • Workload and Family Life • Training and Development • Salary • Physical Security
6	Baier (2018) [24]	Germany	1,101 EMS-workers	54.6%	NR	• Job Dissatisfaction • Burnout • Dissatisfaction with professional status
7	Crowe (2018) [25]	USA	2153 EMTs and paramedics	8.1%	NR	• Burnout
8	Cash (2019) [26]	USA	2815 EMS professionals	7%	NR	• workplace incivility
9	Rivard (2020) [27]	USA	18,285 EMS professionals	6.1%	NR	• Financial Dependence on Extra Work • Job Dissatisfaction
10	Hendrickson (2022) [28]	USA	200 First Responders	40.6	NR	• COVID-19 Related Occupational Stressors • Demoralization • PTSD Symptoms
11	Mousavi (2022) [29]	Iran	315 EMTs	NR	12.54 (4.52)	• Ethical work climate • Contract type (temporary) • Younger age
12	Srikanth (2022) [30]	USA	123 EMTs	59%	NR	• High levels of stress • lack of support from managers • Work experience (under 3 years) • High job demands
13	Herttuainen (2023) [31]	Finland	152 paramedics	23%	NR	• Chronic Stress • Organizational Inequity and Leadership Challenges • job's impact on social life, health, and personal well-being (e.g., fatigue, shift work, risk of injury)
14	Nordquist (2023) [32]	Finland	427 paramedics	21.6%	NR	• higher level of critical incident stress
15	Aras (2024) [33]	Turkey	401 EMTs & paramedics	NR	3.24 (1.08)	• Burnout • Low job satisfaction • Work experience (6–9 years) • lacked knowledge about COVID-19, • no COVID-19 training • experienced the loss of a healthcare worker due to COVID-19
16	Gage (2024) [34]	USA	29,671 EMS professionals	7.47%	NR	• Low job satisfaction • Stress • Covid-19 • Higher education level • Younger age • Pursuing further education
17	Hulkkonen (2024) [35]	Finland	433 paramedics	21%	NR	• Work Experience (more than 10 years) • Lower salutogenic health scores • Decreased work ability
18	Kaplan (2024) [8]	USA	433 EMS professionals	NR	2.78 (0.92)	• Low Emotional Intelligence • Perceived Stress • Poor Physical Health • Low Income • Work experience (15–20 years)
19	Remington (2024) [12]	USA	1,314 EMS professionals	NR	2.63 (1.63)	• higher perceived threat of COVID-19 • Lower perceived threat of COVID-19 to the public • lower sense of emotional safety • high work experience • younger age • gender (male)
20	Suokonautio (2024) [36]	Finland	616 EMS personnel	17.7%	NR	• Longer work experience • Education Level (Personnel with a master's degree) • Role Identity
21	Dao-Tran (2025) [37]	Australia	492 ambulance personnel	70%	NR	• lower job satisfaction • Mental Health Symptoms • Lack of Supervisor and Colleague Support
22	Gage (2025) [38]	USA	33,414 EMS clinicians	NR	NR	• Lowe job satisfaction • Nature of work • Pay • Promotion
23	Hofmann (2025) [3]	Germany	814 ambulance staff	64%	NR	• Unfavorable Working Conditions • lower job satisfaction • Lower organizational commitment
24	Homaei (2025) [39]	Iran	203 EMTs	47.8%	NR	• Workplace Incivility
25	Kamholz (2025) [40]	USA	30,762 EMS clinicians	10.1%	NR	• Organizational culture (Adhocracy Culture, Hierarchy Culture, Market Culture) • lower job satisfaction
26	Meacham (2025) [41]	Australia	596 paramedics	NR	2.50 (0.97)	• role overload • low family support
27	Powell (2025) [42]	USA	1,838 EMS clinicians	17%	NR	• Burnout

*TI* Turnover Intention

*NR* not reported

### Risk of bias in included studies

The methodological quality assessment of the 27 selected studies using the JBI tool indicated good quality. Most studies (83%) were rated as high quality. Only five studies, with one to two instances of bias, were of acceptable quality for inclusion. No study was excluded due to low quality (detailed assessment results are in Appendix Table S1).

### Meta-analysis results

**Fig. 2:**
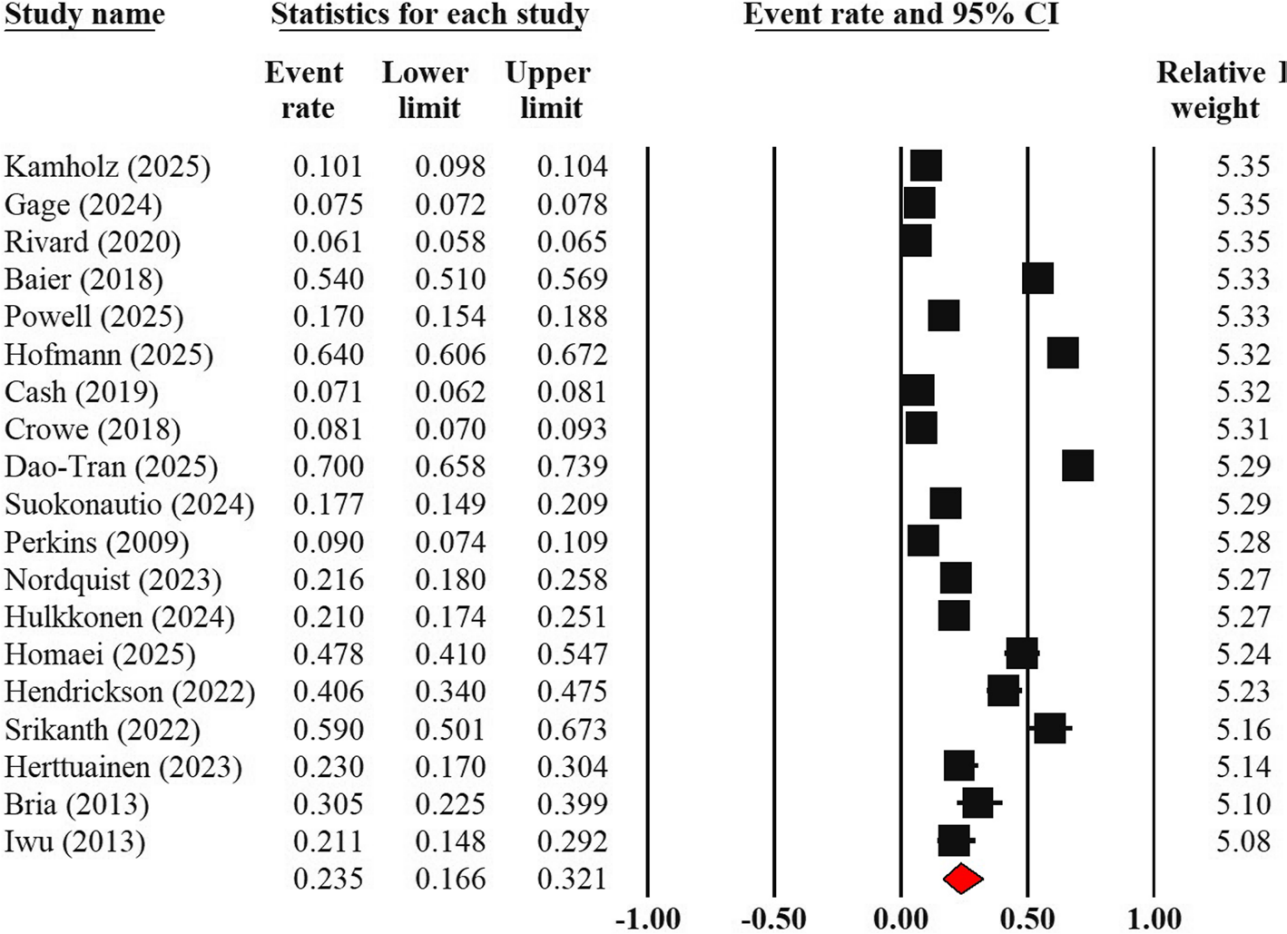
Forest plot of pooled prevalence of turnover intention

Among the 27 studies, 19 reporting the prevalence of turnover intention, with a total of 91,320 participants, were included in the meta-analysis (Fig. 2). Based on the random-effects model, the pooled prevalence of turnover intention among EMS professionals was 23.5% (95% Confidence Interval [CI]: 16.6% to 32.1%). The heterogeneity analysis revealed a very high level of variance (Cochran's Q = 5274.6, *p* < 0.001). The I^2^ statistic was 99.6%, indicating substantial and significant heterogeneity.

The sensitivity analysis showed the removal of any single study did not significantly impact the final pooled prevalence, with new estimates fluctuating within a narrow range of 21.7% to 25.1%, demonstrating the result's robustness.

**Fig. 3:**
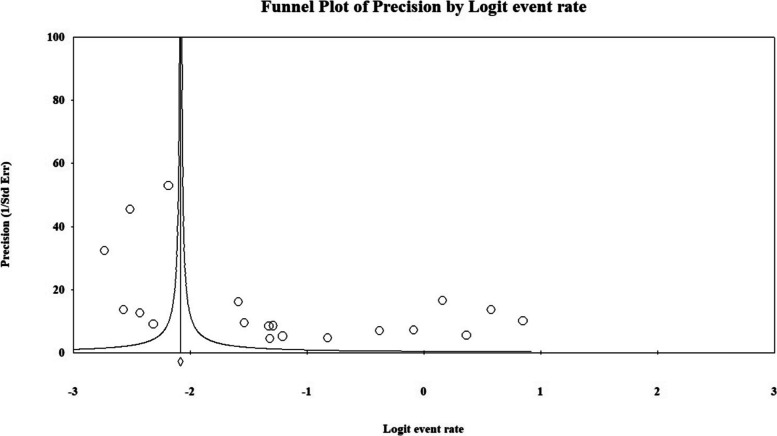
Funnel plots assessing potential publication bias

A visual inspection of the funnel plot showed asymmetry (Fig. 3), and Egger's linear regression test statistically confirmed this asymmetry (*p* = 0.008). To investigate the potential impact of unpublished studies, the Duval and Tweedie trim and fill method was applied. This analysis did not identify or impute any missing studies, and consequently, the adjusted pooled estimate did not differ from the initial estimate. It should be noted, however, that the reliability of this sensitivity analysis may be limited given the extremely high observed heterogeneity.

To identify potential sources of heterogeneity, subgroup analyses were performed based on pre-specified variables (Table 2). The key findings are as follows:

- Sample Size: A highly significant difference was observed between studies with small samples (< 1000 participants) and large samples (≥ 1000 participants) (*p* < 0.001). The pooled prevalence in the small-sample group (36.4%) was substantially higher than in the large-sample group (11.7%).
- Geographic Region: A significant difference was found among geographic regions (*p* < 0.001). The pooled prevalence varied significantly between North America (13.4%), Europe (31.6%), and other regions (45.7%).
- Country Income Level: The prevalence in low- and middle-income countries (33.4%) was considerably higher than in high-income countries (22.5%), but this difference was not statistically significant (*p* = 0.504).
- Publication Year and COVID-19 Pandemic: While the prevalence in more recent studies (2021–2025) (29.6%) was higher than in older studies (2010–2020) (15.2%), this difference was not statistically significant (*p* = 0.134). Similarly, no significant difference was observed between data collected before and during or after the COVID-19 pandemic (*p* = 0.525).

**Table 2:** Subgroup’s analysis of turnover intention

*P*-value	I^2^	95% CI		Pooled Prevalence	Number of studies	Subgroup	
		Upper	Lower				
0.001	99.0	17.1	10.4	13.4	9	America	Region
	98.8	48.3	18.5	31.6	7	Europe	
	97.8	71.9	21.7	45.7	3	Other	
0.405	99.7	31.3	15.6	22.5	17	HICs	Income group
	95.4	62.6	13.0	33.4	2	LMICs	
0.000	99.6	17.3	0.08	11.7	8	Large (≥ 1000)	Sample size
	98.4	50.9	24.0	36.4	11	Small (≤ 1000)	
0.134	99.6	42.0	19.7	29.6	12	2021–2025	Publication time
	99.6	32.3	0.06	15.2	7	2010–2020	
0.525	99.7	41.3	0.08	19.8	8	Before	COVID-19
	99.6	37.1	18.0	26.4	11	After	

### Factors influencing turnover intention

The qualitative content analysis of the 27 selected studies revealed a set of predictive factors for turnover intention, classified based on the expanded JD-R model into four main categories: "Job Demands," "Job Resources," "Personal Resources and Demands," and "Contextual Factors" (Table 3).

**Table 3:** Summary of factors influencing turnover intention and their reporting frequency

Category	Main Factors (Frequency of report in studies)
Job Demands	Job Stress (8); Burnout (5); High Workload (4); Poor Work Environment Quality (5); Type of Duties & Ratio of Emergency Calls (2); Job Absenteeism (2)
Job Resources	Job Satisfaction (11); Salary & Benefits (6); Organizational Issues (5); Opportunities for Growth & Promotion (4); Lack of Supervisor/Colleague Support (2); Organizational Commitment (2); Other (Job enjoyment, Nature of work, Professional identity) (3)
Personal Resources & Demands	Personal Health Status (Physical/Mental) (8); Work-Family Conflict (2); Other Individual Factors (Emotional intelligence, Morale, Intention to change career, etc.) (5)
Contextual Factors	Work Experience (7); Age (4); Education Level (2); Gender (1); Type of Employment Contract (1)

#### Job demands

Job demands—the physical, social, or organizational aspects of a job requiring sustained effort—were identified as primary drivers of turnover intention. Job stress was the most frequently cited predictor (eight studies), encompassing stress from critical incidents, chronic work-related stress, and COVID-19 pandemic pressures [8, 21, 28, 30–34]. Burnout (five studies), particularly its dimensions of emotional exhaustion and depersonalization [22, 24, 25, 33, 42], and high workload, defined by long hours and role overload (four studies), also showed a significant association with turnover intention [21–23, 41]. Other important factors in this category included poor work environment quality [3], experiences of incivility [26, 39], lack of emotional safety [12], and frequent job absenteeism [20], all of which contribute to increased psychological strain and the desire to leave the profession.

#### Job Resources

Job resources are the supportive and motivational aspects of a job that help in achieving goals and reducing stress. The review's results indicate that a lack of these resources is a strong predictor of turnover intention. Low job satisfaction was the central factor in this category, directly linked to increased turnover intention in eleven studies [3, 6, 20, 21, 24, 27, 33, 34, 37, 38, 40]. Inadequate salary and benefits [8, 20, 21, 23, 27, 38] and a lack of opportunities for professional growth [3, 20, 23, 33] were also identified as critical economic and motivational factors. Organizational issues such as poor organizational culture, perceived injustice, and ineffective leadership [21, 23, 29, 31, 40], along with a lack of support from supervisors and colleagues [30, 37], lead to weakened organizational commitment [3, 19] and an increased likelihood of departure. Job-related factors, such as professional identity and satisfaction, were also reported in four studies [23, 24, 36, 38].

#### Personal demands and resources

This category addresses individual characteristics and personal life conditions that interact with the work environment. This section was well done but could be easier to read by bifurcating some sentences for better reader digestion. Personal health status, particularly poor physical and mental health, symptoms of Post-Traumatic Stress Disorder (PTSD), and reduced work ability, was reported as a crucial determinant in eight studies [8, 19, 20, 28, 31, 34, 35, 37]. Work-family conflict (two studies), the negative interference of job requirements with personal life, was also identified as a significant stressor disrupting life balance and encouraging departure [21, 23]. Other reported individual factors included low emotional intelligence [8], diminished morale [28], lack of family support [41], and an intention to pursue further education or change career paths [21, 34], all influencing the decision to stay or leave.

#### Contextual (Demographic) factors

Demographic factors also showed varied associations with turnover intention. Work experience (seven studies) was the most frequently reported factor, though with contradictory results; some studies indicated that employees with less experience (< 5 years) had higher turnover intention [30, 33], while others found that more experienced employees (> 10 years) had a greater desire to leave [8, 12, 21, 35, 36]. Age generally showed younger employees had higher turnover intention [12, 29, 34]. In more limited cases, higher education level [34, 36], male gender [12], and temporary employment contract [29] were also associated with an increased intention to leave among EMS professionals.

## Discussion

As the first comprehensive global systematic review and meta-analysis on the topic, this study investigated the prevalence of turnover intention and its determinants among pre-hospital emergency professionals. The primary finding is a pooled prevalence of 23.5% for turnover intention, indicating that approximately one in four EMS professionals are seriously considering leaving their profession. This is a sobering and alarming figure that poses a significant threat to the sustainability and effectiveness of pre-hospital healthcare systems worldwide. On a qualitative level, the narrative synthesis, framed within the Job Demands–Resources (JD-R) model, consistently identified low job satisfaction and high job stress as central drivers of this intention. Although the estimated prevalence is lower than that reported for some comparable groups such as emergency nurses (45%) [10] or emergency physicians in China (55.2%) [11], a rate of 23.5% still signals a potential crisis for this indispensable workforce.

However, a precise interpretation of this pooled estimate requires careful consideration of the exceptionally high statistical heterogeneity across the included studies (*I*^*2*^ > 99%). As emphasized in the Cochrane Handbook, when studies differ substantially, quoting a single average effect may be misleading [43]. In such cases, the focus of interpretation should shift from the numerical centrality of the pooled estimate to the patterns of variation itself. Accordingly, while the 23.5% figure serves as a useful heuristic to convey the global scale of the problem, it should not be construed as a precise or generalizable prevalence rate. The true insight lies in the extreme variability observed across studies from 6.1% to 70%. This variability is not merely statistical “noise”; it is a substantive finding that underscores the context-dependent nature of turnover intention among EMS professionals.

Three key analytical findings help explain the sources of this heterogeneity and illustrate why a single global estimate can obscure more than it reveals:

First, publication bias may have inflated the pooled prevalence. The analysis confirmed funnel plot asymmetry (Egger’s test, p = 0.008), suggesting that studies reporting higher turnover intention were more likely to be published. This implies the true underlying prevalence might be somewhat lower than 23.5%, underscoring the need for greater reporting of studies with null or low-prevalence findings.

Second, the geographical composition of the evidence base introduces a counteracting bias toward underestimation. The majority of included studies originated from North America, where the prevalence was lowest (13.4%), while regions with markedly higher rates, such as Europe (31.6%) and other areas (45.7%), were underrepresented. This imbalance likely conceals the full severity of the issue in non-North American settings.

Third, the stark contrast between small- and large-scale studies reveals a fundamental methodological divide. Small studies (n < 1,000) reported a pooled prevalence of 36.4%, more than triple that of large-scale studies (11.7%). This discrepancy likely stems from differences in sampling: large studies often draw from national datasets and reflect workforce-wide trends, whereas small studies frequently focus on single organizations or crisis-affected units, yielding locally intense but less generalizable estimates. This pattern may also be amplified by publication bias, as dramatic findings from small samples are more likely to attract publication [44, 45].

The analysis of predictive factors based on the JD-R model provides a deeper understanding of the crisis's roots. The narrative synthesis shows that an imbalance between high job demands and inadequate job resources is the core driver of turnover intention. Among these, low job satisfaction, the most frequently identified factor, appears to be the culmination of other resource deficits. Job satisfaction, as an indicator of occupational well-being, is directly influenced by work environment conditions, social support, and mental health [4]. When employees face inadequate salary, lack of support from supervisors, a poor organizational culture, and limited growth opportunities, these factors ultimately result in job dissatisfaction. For example, hierarchical organizational cultures or negative interactions like workplace incivility can erode trust, damage professional relationships, and create a toxic work environment, directly increasing turnover intention [39, 40]. Conversely, peer support can play a protective role through supportive post-incident discussions and professional validation [32].

An important finding is the dual nature of job satisfaction among EMS professionals. Despite intense physical, emotional, and time pressures, these individuals often derive high satisfaction from intrinsic factors of their work, such as helping others, which strengthens retention [38]. However, the evidence clearly indicates that the primary driver toward turnover is dissatisfaction with extrinsic factors, particularly low pay and the absence of a career progression path [2, 3, 6, 38]. This situation, where high effort is not compensated with commensurate rewards, is well-explained by the effort-reward imbalance theoretical framework [5]. Since organizational factors have high potential for improvement, interventions should focus on this area. Organizations can respond by revising compensation policies and, more importantly, developing innovative career pathways. Introducing new roles, such as the "paramedic practitioner" or specialized response units, can provide meaningful opportunities for clinical growth, enhance job satisfaction, and ultimately help reduce turnover intention [7, 9].

Stress and burnout, the most common job demands identified in this profession, are directly tied to the nature of the work. Continuous exposure to distressing scenes, high responsibility under uncertainty, and a heavy workload impose severe psychological and emotional demands on professionals [4, 5]. Studies show that pre-hospital emergency staff experience higher levels of stress and burnout than many other occupations, including emergency physicians [5, 6], and burnout has been cited as a significant issue that can negatively impact EMS recruitment and retention [25]. While altering the inherently stressful nature of this job is difficult, accepting burnout as an "inevitable" consequence can hinder preventive organizational measures. Burnout is less an individual failure and more a consequence of correctable organizational conditions [7]. Therefore, a shift in focus from the individual to the system is essential. Communication campaigns that frame burnout as a systemic challenge, alongside organizational leaders who promote a positive work environment, play a key role in reducing burnout and increasing staff retention [9, 13, 42]. At a practical level, measures such as improving access to proper patient-handling equipment can directly prevent physical injuries and reduce physical strain [7].

The analysis of demographic factors revealed notable associations. The relationship between work experience and turnover intention appears to follow a U-shaped pattern. New employees may experience disillusionment due to a "reality shock”, the gap between academic expectations and field realities. On the other hand, experienced staff may consider leaving due to cumulative burnout, chronic stress, and the job's long-term effects on their physical and mental health [6]. Regarding age, evidence suggests younger employees are at greater risk of turnover due to factors like high expectations for advancement and less developed coping skills [10, 33]. Personal health also plays a vital role. Irregular shift work disrupts the circadian rhythm, leading to health problems such as cardiovascular disease and sleep disorders [35]. The evidence confirms that poor physical and mental health, especially sleep disturbances, are strong predictors of turnover intention [6].

### Strengths and limitations

The primary strength of this research is its comprehensiveness as the first global systematic review and meta-analysis in this field, based on data from over 129,000 participants. The use of advanced statistical methods adds to the findings' validity. Nevertheless, this study has several limitations. The very high statistical heterogeneity warrants caution in interpreting the pooled prevalence, although subgroup analyses explained a portion of this variance. Another limitation is the imbalanced geographical distribution of studies, with a focus on the United States that may limit generalizability. The presence of publication bias also suggests that studies with low prevalence were likely under-published, which could have influenced the overall estimate. Finally, the predominantly cross-sectional nature of the included studies precludes definitive conclusions about cause-and-effect relationships.

### Research recommendations

To overcome these limitations, future research should be directed along several key paths. First, longitudinal studies are needed to track the causal relationships between predictive factors and turnover intention over time. Second, qualitative studies exploring the lived experiences of professionals in various cultural contexts are essential. Third, research must expand beyond North America to cover less-studied regions to paint a more comprehensive global picture. Fourth, interventional studies are needed to evaluate the effectiveness of organizational programs in reducing turnover intention.

### Policy recommendations

The study's findings offer direct implications for organizational leaders and health policymakers. As turnover among EMS professionals is influenced by a complex interplay of variables, a comprehensive approach is required to recruit and retain a sufficient workforce [6]. To reduce turnover, policies are needed that simultaneously address organizational conditions and structural reforms.

- At the Organizational Level (for Managers and Leaders): The paradigm must shift from a reactive approach (replacing staff) to a proactive one based on investing in job resources. Here, managers focus on operational support and workflow, while leaders shape a positive culture and strategic vision [30, 40]. Specific actions can include fostering supportive leadership [10, 31, 33], establishing mentorship programs [30], revising compensation packages to create an effort-reward balance [5, 30], and providing meaningful professional development opportunities [33]. Concurrently, managing job demands through stress management programs, resilience training, and ergonomic shift design is vital [5, 30, 33].
- At the Macro Level (for Health Policymakers): The issue of paramedic retention is a systemic problem requiring solutions at the national level from health policymakers, defined as the bodies responsible for establishing health system standards and legislation. Key policy actions could include officially recognizing the profession as high-risk [10], providing early retirement options [5], creating standardized career advancement pathways [2], developing national standards for salaries and benefits [5], and funding specialized mental health services for EMS professionals [33].

## Conclusion

Turnover intention among pre-hospital emergency professionals is a serious and pervasive global challenge that threatens the sustainability of this critical healthcare sector. The findings indicate this phenomenon is less an isolated individual decision and more a predictable response to a work environment where intense demands are met with inadequate resources. Effectively combating this crisis requires a coordinated, dual approach: actions by organizational leaders to strengthen job resources, and structural reforms by health policymakers to elevate professional standing and support the well-being of this irreplaceable workforce.

## Supplementary Information

Supplementary Material 1.

Supplementary Material 2.

## Data Availability

No datasets were generated or analysed during the current study.
