# Supplementary material for: Turnover intention and its predictors among Emergency Medical Services (EMS) professionals: a systematic review and meta-analysis
Source: Scand J Trauma Resusc Emerg Med. 2026 Jan 26;34:42. doi: 10.1186/s13049-026-01567-8 (PMC12918125; doi:10.1186/s13049-026-01567-8)
Supplement: Supplementary file 1 — Supplementary Material 1. [file 13049_2026_1567_MOESM1_ESM.docx]

Table S1. Quality assessment of included articles

| Score | Appropriate Statistical Analysis | Valid and Reliable Measure of Outcome | Strategies for Dealing with Confounding Factors | Confounding Factors Identified | Objective and Standard Measure of Condition | Valid and Reliable Measure of Exposure | Subjects and Setting Detailed Description | Inclusion Criteria | Author(s)/ Year |  |
| --- | --- | --- | --- | --- | --- | --- | --- | --- | --- | --- |
| 8 | ✓ | ✓ | ✓ | ✓ | ✓ | ✓ | ✓ | ✓ | Powell (2025) | 1 |
| 8 | ✓ | ✓ | ✓ | ✓ | ✓ | ✓ | ✓ | ✓ | Meacham (2025) | 2 |
| 8 | ✓ | ✓ | ✓ | ✓ | ✓ | ✓ | ✓ | ✓ | Kamholz (2025) | 3 |
| 8 | ✓ | ✓ | ✓ | ✓ | ✓ | ✓ | ✓ | ✓ | Homaei (2025) | 4 |
| 8 | ✓ | ✓ | ✓ | ✓ | ✓ | ✓ | ✓ | ✓ | Hofmann (2025) | 5 |
| 8 | ✓ | ✓ | ✓ | ✓ | ✓ | ✓ | ✓ | ✓ | Gage (2025) | 6 |
| 8 | ✓ | ✓ | ✓ | ✓ | ✓ | ✓ | ✓ | ✓ | Dao-Tran (2025) | 7 |
| 8 | ✓ | ✓ | ✓ | ✓ | ✓ | ✓ | ✓ | ✓ | Suokonautio (2024) | 8 |
| 8 | ✓ | ✓ | ✓ | ✓ | ✓ | ✓ | ✓ | ✓ | Remington (2024) | 9 |
| 8 | ✓ | ✓ | ✓ | ✓ | ✓ | ✓ | ✓ | ✓ | Kaplan (2024) | 10 |
| 8 | ✓ | ✓ | ✓ | ✓ | ✓ | ✓ | ✓ | ✓ | Hulkkonen (2024) | 11 |
| 8 | ✓ | ✓ | ✓ | ✓ | ✓ | ✓ | ✓ | ✓ | Gage (2024) | 12 |
| 7 | ✓ | ✓ | ✓ | ✓ | ✓ | ✓ | × | ✓ | Aras (2024) | 13 |
| 8 | ✓ | ✓ | ✓ | ✓ | ✓ | ✓ | ✓ | ✓ | Nordquist (2023) | 14 |
| 6 | × | ✓ | × | ✓ | ✓ | ✓ | ✓ | ✓ | Herttuainen (2023) | 15 |
| 7 | ✓ | ✓ | ✓ | ✓ | ✓ | ✓ | × | ✓ | Srikanth (2022) | 16 |
| 8 | ✓ | ✓ | ✓ | ✓ | ✓ | ✓ | ✓ | ✓ | Hendrickson (2022) | 17 |
| 7 | ✓ | ✓ | × | ✓ | ✓ | ✓ | ✓ | ✓ | Mousavi (2022) | 18 |
| 8 | ✓ | ✓ | ✓ | ✓ | ✓ | ✓ | ✓ | ✓ | Rivard (2020) | 19 |
| 8 | ✓ | ✓ | ✓ | ✓ | ✓ | ✓ | ✓ | ✓ | Cash (2019) | 20 |
| 8 | ✓ | ✓ | ✓ | ✓ | ✓ | ✓ | ✓ | ✓ | Crowe (2018) | 21 |
| 8 | ✓ | ✓ | ✓ | ✓ | ✓ | ✓ | ✓ | ✓ | Baier (2018) | 22 |
| 5 | × | ✓ | × | ✓ | ✓ | × | ✓ | ✓ | Iwu (2013) | 23 |
| 7 | ✓ | × | ✓ | ✓ | ✓ | ✓ | ✓ | ✓ | Bria (2013) | 24 |
| 7 | ✓ | ✓ | ✓ | ✓ | ✓ | × | ✓ | ✓ | Perkins (2009) | 25 |
| 8 | ✓ | ✓ | ✓ | ✓ | ✓ | ✓ | ✓ | ✓ | Chapman (2009) | 26 |
| 8 | ✓ | ✓ | ✓ | ✓ | ✓ | ✓ | ✓ | ✓ | Blau (2009) | 27 |
