# Supplementary material for: Turnover intention and its predictors among Emergency Medical Services (EMS) professionals: a systematic review and meta-analysis
Source: Scand J Trauma Resusc Emerg Med. 2026 Jan 26;34:42. doi: 10.1186/s13049-026-01567-8 (PMC12918125; doi:10.1186/s13049-026-01567-8)
Supplement: Supplementary file 2 — Supplementary Material 2. [file 13049_2026_1567_MOESM2_ESM.docx]

WOS

((((TS=(personnel turnover )) OR TS=(intention to leave)) OR TS=(intention to quit)) OR TS=(turnover intention)) OR TS=(retention )

(((((((((TS=(emergency responders)) OR TS=(paramedic )) OR TS=(emt paramedics)) OR TS=(emergency medical technicians)) OR TS=(emergency medical services)) OR TS=(first responders)) OR TS=(lifesaver)) OR TS=(ambulance crew )) OR TS=(Emergency Medical personnel )) OR TS=(Emergency Medical professional )OR TS=(Ambulance Personnel ) OR TS=(EMS Providers ) OR TS=(Prehospital Emergency Technicians)

#1 AND #2

Pubmed

(((((personnel turnovers[MeSH Terms]) OR (turnover intention[Title/Abstract])) OR (intention to leave[Title/Abstract])) OR (intention to quit[Title/Abstract])) OR (turnover[Title/Abstract])) AND ((((((((((((((((emergency responders[Title/Abstract]) OR (paramedic[Title/Abstract])) OR (emt paramedics[Title/Abstract])) OR (emergency medical technicians[Title/Abstract])) OR (emergency medical services[Title/Abstract])) OR (first responders[Title/Abstract])) OR (lifesaver[Title/Abstract])) OR (ambulance crew[Title/Abstract])) OR (Emergency Medical personnel[Title/Abstract])) OR (Emergency Medical professional[Title/Abstract])) OR (Ambulance Personnel[Title/Abstract])) OR (EMS Providers[Title/Abstract])) OR (Prehospital Emergency Technicians[Title/Abstract])) OR (paramedics[MeSH Terms])) OR (emt paramedics[MeSH Terms])) OR (emergency medical technicians[MeSH Terms])) Filters: from 2000 – 2025

scopus

( ( TITLE-ABS-KEY ( personnel turnovers ) OR TITLE-ABS-KEY ( turnover intention ) OR TITLE-ABS-KEY ( intention to leave ) OR TITLE-ABS-KEY ( intention to quit ) OR TITLE-ABS-KEY ( turnover ) OR TITLE-ABS-KEY ( retention ) ) ) AND ( ( TITLE-ABS-KEY ( emergency responders ) OR TITLE-ABS-KEY ( paramedic ) OR TITLE-ABS-KEY ( emt paramedics ) OR TITLE-ABS-KEY ( emergency medical technicians ) OR TITLE-ABS-KEY ( emergency medical services ) OR TITLE-ABS-KEY ( first responders ) OR TITLE-ABS-KEY ( lifesaver ) OR TITLE-ABS-KEY ( ambulance crew ) OR TITLE-ABS-KEY ( Emergency Medical personnel ) OR TITLE-ABS-KEY ( Emergency Medical professional ) OR TITLE-ABS-KEY ( Ambulance Personnel ) OR TITLE-ABS-KEY ( EMS Providers ) OR TITLE-ABS-KEY ( Prehospital Emergency Technicians ) ) ) AND PUBYEAR > 1999 AND PUBYEAR < 2026 AND ( LIMIT-TO ( LANGUAGE , "English" ) ) AND ( LIMIT-TO ( SRCTYPE , "j" ) )
